# Case validation of bloodstream infections with an antibiotic-resistant organism

**DOI:** 10.1017/ash.2024.336

**Published:** 2024-09-16

**Authors:** Jennifer Ellison, Blanda Chow, Andrea Howatt, Logan Armstrong, Ted Pfister, Zhe Lu, Kathryn Bush

**Affiliations:** Infection Prevention & Control, Alberta Health Services; Alberta Health Services

## Abstract

**Background:** Bloodstream infections (BSIs) are an important cause of morbidity and mortality in severely ill patients, contributing to increased length of hospital stay and higher cost of care. Alberta Health Services Infection Prevention and Control (IPC) conducts inpatient surveillance of new episodes of BSIs with methicillin-resistant Staphylococcus aureus (MRSA), vancomycin-resistant enterococci (VRE) or carbapenemase-producing organisms (CPO) in 112 acute care facilities. A case-finding process was undertaken to verify the accuracy of BSI data entry. **Methods:** All positive MRSA, VRE or CPO blood cultures in 2021 were linked to the Inpatient Discharge Abstract Database (DAD) and the National Ambulatory Care Reporting System (NACRS) to identify new cases during acute care admissions. The results were then compared to surveillance records captured by infection control professionals (ICPs). Cases with unmatched culture date and/or encounter date and cases not identified by ICPs were screened by the study team with final decision made by ICPs. Results were analyzed by ARO and by % increase in number of surveillance records. **Results:** The laboratory linkage identified 286 new cases. Comparing to surveillance records (n = 248) captured by ICPs, 137 (57.3%) had matching collection dates and encounter dates, 85 (35.6%) had close matches on collection dates and encounter dates, 17 (7.1%) records had either matching collection dates or encounter dates, and 1 (0.4%) record did not have any matches on dates. There were 46 records identified in the laboratory data that were not in the surveillance system and 8 records that were in the surveillance system but not matched to the laboratory data. After review, 22 Surveillance records had data entry errors (1 CPO BSI, 20 MRSA BSI, and 1 VRE BSI), and there were 14 BSI records found to be missing (13 MRSA BSI, 1 VRE BSI). This represents a 6% increase in MRSA BSI and a 3% increase in VRE BSI identified in 2021 and no increase in CPO BSI. **Conclusions:** A laboratory validation to determine if BSIs with an ARO were missed during routine IPC surveillance identified a small proportion of missed bloodstream infections. The most common reason for the miss was admission through the emergency department with multiple blood cultures collected during a single admission. These results will be shared with the Infection Control program to facilitate correct BSI capture.

